# Gonadotropin-Releasing Hormone (GnRH) Neuron Potassium Currents and Excitability in Both Sexes Exhibit Minimal Changes upon Removal of Negative Feedback

**DOI:** 10.1523/ENEURO.0126-21.2021

**Published:** 2021-07-03

**Authors:** R. Anthony DeFazio, Suzanne M. Moenter

**Affiliations:** 1 Department of Molecular & Integrative Physiology, University of Michigan, Ann Arbor, Michigan 48109; 2 Department of Obstetrics and Gynecology, University of Michigan, Ann Arbor, Michigan 48109; 3 Department of Internal Medicine, University of Michigan, Ann Arbor, Michigan 48109

**Keywords:** action potential, castration, GnRH, potassium current, sex, sex steroids

## Abstract

Gonadotropin-releasing hormone (GnRH) drives pituitary secretion of luteinizing hormone and follicle-stimulating hormone, which in turn regulate gonadal functions including steroidogenesis. The pattern of GnRH release and thus fertility depend on gonadal steroid feedback. Under homeostatic (negative) feedback conditions, removal of the gonads from either females or males increases the amplitude and frequency of GnRH release and alters the long-term firing pattern of these neurons in brain slices. The neurobiological mechanisms intrinsic to GnRH neurons that are altered by homeostatic feedback are not well studied and have not been compared between sexes. During estradiol-positive feedback, which is unique to females, there are correlated changes in voltage-gated potassium currents and neuronal excitability. We thus hypothesized that these same mechanisms would be engaged in homeostatic negative feedback. Voltage-gated potassium channels play a direct role in setting excitability and action potential properties. Whole-cell patch-clamp recordings of GFP-identified GnRH neurons in brain slices from sham-operated and castrated adult female and male mice were made to assess fast and slow inactivating potassium currents as well as action potential properties. Surprisingly, no changes were observed among groups in most potassium current properties, input resistance, or capacitance, and this was reflected in a lack of differences in excitability and specific action potential properties. These results support the concept that, in contrast to positive feedback, steroid-negative feedback regulation of GnRH neurons in both sexes is likely conveyed to GnRH neurons via mechanisms that do not induce major changes in the biophysical properties of these cells.

## Significance Statement

The pattern of activity of gonadotropin-releasing hormone (GnRH) neurons is crucial to reproductive success in both males and females. Direct comparison of GnRH neurons from mice of both sexes during negative feedback and after gonadectomy revealed few differences in potassium currents, excitability, and action potential properties. These results support the hypothesis that neurons presynaptic to GnRH neurons communicate negative feedback to these cells in a manner that does not alter their intrinsic biophysical properties.

## Introduction

The episodic release of gonadotropin-releasing hormone (GnRH) from the brain is key to successful reproduction in both sexes. GnRH regulates pituitary release of luteinizing hormone (LH) and follicle-stimulating hormone, which activate gonadal functions including the production of sex steroids. These steroids feed back to regulate the pattern of GnRH release and pituitary response to GnRH. Aspects of this feedback system are well established as sexually differentiated; for example, estradiol-positive feedback is exclusive to females under normal physiologic conditions and induces the preovulatory surge of GnRH (Docke and Dorner, 1965; Sarkar et al., 1976; Moenter et al., 1991). Other aspects appear similar between the sexes, including homeostatic negative feedback that regulates episodic GnRH release and is critical for maintaining most reproductive processes in both males and females. Gonadectomy increases GnRH and LH release (Leipheimer et al., 1985; Levine et al., 1985; Karsch et al., 1987; Caraty and Locatelli, 1988; Condon et al., 1988; Jackson and Kuehl, 2000; Czieselsky et al., 2016). The main circulating sex steroids providing homeostatic feedback are sexually differentiated, specifically estradiol and progesterone in females, depending on the stage of the reproductive cycle, and testosterone in males. The differences in circulating steroids are reduced in part in the brain by the conversion of testosterone to estradiol (Fisher et al., 1998; Sharma et al., 1999), and the efficacy of androgens and estrogens in eliciting negative feedback in males varies with species (Plant, 1982; Levine and Duffy, 1988; Tilbrook et al., 1999).

Regulation of episodic GnRH release is a key potential intervention point for the very different goals of reversibly inhibiting reproduction for contraception and ameliorating central infertility. Despite a similar hormonal response to the removal of peripheral sex steroid feedback by gonadectomy, whether or not the underlying intrinsic changes in GnRH neurons that lead to these increases are sexually differentiated is unknown. Studies in females have focused on the mechanisms underlying estradiol-positive feedback that is critical for inducing ovulation; many of these have compared gonadectomized (GDX) animals with open feedback loops to those with specific steroid replacement (Wagner et al., 2001; Christian and Moenter, 2010; Dror et al., 2013; Liu et al., 2017; Adams et al., 2018b, 2019; Wang et al., 2019). These studies have revealed changes in both intrinsic GnRH neuron properties and fast synaptic input to these cells. Studies of homeostatic negative feedback suggest that the firing pattern of GnRH neurons varies during the reproductive cycle in females and when steroid feedback is disrupted by gonadectomy in both sexes (Pielecka et al., 2006; Pielecka and Moenter, 2006; Silveira et al., 2017). There are no direct comparisons, however, of GnRH neuron intrinsic properties between intact males and females or how these are affected by gonadectomy.

A basic measure of the intrinsic biophysical properties of neurons is their excitability, defined as the number of action potentials generated in response to varying current injections. In females, this varies with cycle stage and with induction of daily LH surges by estradiol in ovariectomized mice (Adams et al., 2018b, 2019), but this parameter has not been characterized in males. Voltage-gated potassium channels are widely recognized as regulators of excitability (Johnston et al., 2010) and have been targeted clinically. For example, blocking a fraction of potassium channels with 4-aminopyridine (4AP) increases the excitability of motor neurons in amyotrophic lateral sclerosis and multiple sclerosis and provides relief from the symptoms of these diseases (Bakirtzis et al., 2018; Peikert et al., 2019). In many neurons, 4AP increases input resistance and diminishes the ability of voltage-gated potassium channels to blunt action potential firing and/or the membrane potential changes in response to synaptic inputs (Hoffman, 2013; DeFazio et al., 2019). Here we tested the hypotheses that voltage-gated potassium currents are reduced and the excitability of GnRH neurons are increased by gonadectomy. While removing negative feedback increases GnRH and LH in both sexes, it is possible that there are sex-dependent mechanistic differences underlying this increase; thus, we examined both males and females.

## Materials and Methods

All chemicals were acquired from Sigma-Aldrich, unless otherwise noted.

### Animals

Mice expressing GFP under control of the GnRH promoter [Tg(GnRH1-EGFP)51Sumo MGI:6158457, GnRH-GFP mice; catalog #033639, The Jackson Laboratory] were propagated in our colony. All mice had *ad libitum* access to Harlan 2916 chow and water, and were held at 21–23°C on a 14 h light/10 h dark cycle with lights on at 3:00 A.M. Eastern Standard Time (EST). Adult mice 82–146 d old were ovariectomized (OVX; females), orchidectomized (ORX; males), or sham operated under isoflurane anesthesia with bupivacaine as a local analgesic. Studies were performed 5–7 d after surgery. In sham-operated females, estrous cycles were monitored by vaginal cytology for at least 10 d before experiments and diestrous mice were selected. Body, uterine, and seminal vesicle mass were recorded at the time of brain slice preparation to verify steroid status. The Institutional Animal Care and Use Committee approved all procedures.

### Brain slice preparation

Coronal brain slices (300 μm) containing the preoptic area (POA) were prepared with a vibratome (model VT1200S, Leica Biosystems). Brain slices were obtained between 7:30 and 8:30 A.M. EST, and recordings were obtained between 8:30 A.M. and 2:30 P.M. EST. The brain was rapidly removed and placed in ice-cold sucrose saline solution containing the following (in mm): 250 sucrose, 3.5 KCl, 26 NaHCO_3_, 10 d-glucose, 1.25 Na_2_HPO_4_, 1.2 MgSO_4_, and 3.8 MgCl_2_ (at 350 mOsm). Slices were incubated for 30 min at room temperature (∼21–23°C) in 50% sucrose saline and 50% artificial CSF (ACSF) containing the following (in mm): 135 NaCl, 3.5 KCl, 26 NaHCO_3_, 10 D-glucose, 1.25 Na_2_HPO_4_, 1.2 MgSO_4_, 2.5 CaCl_2_, at 315 mOsm and pH 7.4. Slices were then transferred to 100% ACSF solution at room temperature for 0.5–5 h before recording. All extracellular solutions were bubbled with 95% O_2_/5% CO_2_ throughout the experiments and for at least 30 min before exposure to tissue. One to three recordings were obtained per mouse with a minimum of five mice studied per group.

### Recording solutions and data acquisition

The pipette solution consisted of the following (in mm): 125 K gluconate, 20 KCl, 10 HEPES, 5 EGTA, 0.1 CaCl_2_, 4 MgATP, and 0.4 NaGTP, at 305 mOsm and pH 7.2 with NaOH (this solution is based on the native intracellular chloride concentration in GnRH neurons determined using gramicidin perforated-patch recordings; DeFazio et al., 2002). A 14.5 mV liquid junction potential was negated before each recording (Barry, 1994). During all recordings, slices were continuously superfused at 2 ml/min with carboxygenated ACSF kept at 30–31°C with an inline-heating unit (Warner Instruments). GFP-positive cells were visualized with a combination of infrared differential interference contrast and fluorescence microscopy on an Olympus BX50WI microscope. Recordings were made with an EPC-10 patch-clamp amplifier and a computer running PATCHMASTER software (HEKA Elektronik). For current-clamp experiments, membrane voltage was acquired at 20 kHz and filtered at 10 kHz; for voltage-clamp experiments, current was acquired at 10 kHz and filtered at 5 kHz. Input resistance, series resistance, baseline current, and capacitance were monitored throughout experiments from the membrane current response to a 20 ms, 5 mV hyperpolarizing voltage step from −65 mV to monitor recording quality. All recordings with input resistances <0.5 GΩ, series resistances >20 MΩ, or unstable membrane capacitance were rejected. Results did not depend on the anatomic location of GnRH neurons within the POA.

### Experimental design

Potassium currents, action potential properties, and passive properties were characterized in GFP-identified GnRH neurons in the preoptic area of brain slices prepared from adult gonad intact and castrated mice of both sexes. Castrated mice were studied 5–7 d postsurgery; intact females were in diestrus.

### Voltage-gated potassium current characterization

Potassium currents were isolated pharmacologically during whole-cell voltage-clamp recordings by blocking fast sodium and calcium channels as well as ionotropic receptors for GABA and glutamate [2 μm tetrodotoxin (Tocris Bioscience), 300 μm NiCl_2_, 20 μm d-APV (Tocris Bioscience), 10 μm CNQX, and 100 μm picrotoxin]. Membrane potential was held at −65 mV between voltage-clamp protocols. Series resistance was monitored before compensation using the current response to a 5 mV hyperpolarizing step. Two potassium currents, *I*_A_ (fast inactivating potassium current) and *I*_K_ (slow inactivating potassium current) were distinguished based on voltage dependence and time course. *I*_A_ is a typical, rapidly inactivating potassium current that can be activated at membrane potentials that are hyperpolarized to the threshold for action potential firing. *I*_K_ also displays voltage-dependent inactivation, but this is restricted to more depolarized potentials and has a slower time course of inactivation.

### Activation and inactivation of *I*_A_

Preliminary studies on *I*_A_ showed that a 500 ms prepulse at −40 mV induced full inactivation, and a prepulse at −100 mV for 500 ms completely removed inactivation. These two prepulses were combined with a series of voltage steps to isolate and characterize *I*_A_. To measure total potassium current, a 500 ms prepulse at −100 mV was first applied to remove the inactivation of the fast transient component, followed by a family of voltage steps (500 ms, 10 mV intervals) from −100 to +50 mV, and then a final step to −10 mV for 100 ms. To inactivate *I*_A_, the same step protocol was applied, but with the 500 ms prepulse set at −40 mV to inactivate the fast component while leaving *I*_K_ mostly unchanged, attributable to its very slow inactivation (see below). The fast *I*_A_ component was then isolated by subtracting the family of currents obtained with the −40 mV prepulse from that obtained with the −100 mV prepulse. Activation of *I*_A_ was quantified by measuring the peak current reached during each voltage step in the family (see Analysis section below). Inactivation of *I*_A_ was estimated from the peak current during the final step to −10 mV that followed the family of voltage steps. All protocols for *I*_A_ analysis were leak subtracted using the online –P/8 (average of eight sweeps, one-eighth the size of the voltage step in the opposite direction, from a baseline potential of −65 mV; Bezanilla and Armstrong, 1977).

Time course of inactivation of *I*_A_ was characterized by stepping the membrane potential to −100 mV for 500 ms to remove inactivation, then stepping to the inactivation potential (−40 mV) for 0, 0.5, 1, 2, 4, 8, 16, 32, 64, 128, 256, 512, and 1024 ms, followed by a test pulse at −10 mV to assess the peak current. The noninactivating component during the test pulse after 1024 ms inactivation was subtracted from each of the other traces to isolate the transient current. Similarly, the time course of recovery from inactivation of *I*_A_ was characterized by stepping the membrane potential to −40 mV for 500 ms to fully inactivate *I*_A_, and then stepping to −100 mV for the durations above, followed by a test pulse at −10 mV to assess the peak current. The noninactivating component at 0 ms recovery was subtracted from each of the other traces to isolate the transient current.

To study *I*_K_, a separate set of recordings was required because of the slow inactivation of this component. Indeed, full inactivation and recovery required >10s at +50 and −100 mV, respectively. Cells did not remain stable on repeated exposure to these potentials; thus, more moderate potentials were used to permit estimation of these properties within the command potentials and durations the cells tolerated.

### Activation and inactivation of *I*_K_

To quantify activation, a double prepulse protocol was used: an initial prepulse to −75 mV for 10s was used to remove inactivation from *I*_K_, then a second prepulse to −50 mV for 1s was used to inactivate *I*_A_. After these two prepulses, test pulses of 10 s from −50 to +50 mV in 10 mV increments were used to measure the peak *I*_K_ current. A final step to +50 mV for 100 ms was used following each test pulse to measure inactivation of *I*_K_. The long-duration voltage steps required to characterize this current make traditional online leak subtraction unrealistic. For the activation of *I*_K_, leak currents were subtracted offline using the approach of Kimm and Bean (2014). The shape of the leak response was acquired from the average of 16 steps of −5 mV for 50 ms from the holding potential of −65 mV. Offline, the average was scaled by the voltage difference and subtracted from the portion of the raw current sweep containing the activation test pulses. The leak steps were run immediately before each voltage-clamp protocol. Before each leak acquisition, passive properties were recorded and both slow capacitance and series resistance compensation (50–70%) adjusted.

Time course of inactivation of *I*_K_ was characterized by first removing inactivation by holding at −75 mV for 10s, then *I*_A_ was inactivated by stepping to –50 mV for 1s. This was followed by a variable duration inactivation pulse (0.1, 0.21, 0.42, 0.83, 1.64, 3.25, 6.46, 12.87, 25.68, and 51.29s) at −30 mV, followed by a test pulse at +50 mV. Because inactivation was incomplete even after 51.29 s at −30 mV, this remaining current was not subtracted and the inactivation graphs level off at ∼30%. Recovery from inactivation for *I*_K_ was studied using an inactivating prepulse of +50 mV for 10 s, followed by a variable duration recovery pulse at −80 mV (0.01, 0.03, 0.06, 0.11, 0.2, 0.37, 0.7, 1.35, 2.64, 5.21, 10.34, and 20.59s), a brief pulse to inactivate *I*_A_ (−50 mV for 1 s), and a test pulse at +50 mV for 100 ms. The peak current was plotted as a function of the recovery prepulse duration. Since inactivation was incomplete, the noninactivated current was not subtracted (recovery graphs start at ∼30%).

### Analysis

Current density was calculated by dividing current by capacitance for each cell. To assess the voltage dependence of activation, both *I*_A_ and *I*_K_ were divided by the driving force derived from the Goldman–Hodgkin–Katz (GHK) current equation (Clay, 2000, 2009). To estimate *V*_0.5activation_, *I*_A_ and *I*_K_ were divided by the GHK driving force, normalized to the maximum value, plotted as a function of step potential and fit with the Boltzmann equation, as follows:

$$I(V)=1/1+e^{-kF/RT(V-V_{0.5})}$$

where *V* is the command potential of the step, *V*_0.5_ is the potential at half-maximum, and *k* is the “slope factor” (*k* has no units attributable to the F/RT factor; where F is the Farraday Constant, R is the Gas Constant, and T is absolute temperature). Voltage dependence of inactivation was characterized using the same equation to fit the normalized current measured during the inactivation test pulse.

### Action potential properties

To characterize action potential (AP) properties, current-clamp recordings were obtained in the presence of antagonists of receptors for fast synaptic transmission (20 μm d-APV, 10 μm CNQX, and 100 μm picrotoxin). Cells were maintained at −65 mV by current injection (<50 pA) in current clamp after bridge compensation of series resistance by at least 95%. Current steps (5 pA increments, 500 ms, −10 to +50 pA, 2.5s interval near −65 mV between sweeps) were delivered to test the membrane potential response. The first current step to display an action potential was defined as the rheobase and the first spike analyzed in detail. Action potential threshold was defined as the potential at which the membrane potential slope exceeded 1 V/s. Action potential latency was defined as the time from the start of the current injection to threshold. The rate of rise was defined as the maximum of the voltage trace derivative from threshold to peak. Full-width at half-maximum (FWHM) of the action potential, and afterhyperpolarization (AHP) time and amplitude were measured relative to threshold.

### Statistics

Data are reported as the mean ± SEM, with individual values shown where practical. Statistical comparisons were made using Prism 9 (GraphPad Software). Data were tested for normal distribution with the Shapiro–Wilk test. Tests were chosen appropriate for the experimental design and data distribution, as specified in the Results, and included two sample tests, two-way ANOVA (sex vs gonadal status) with or without repeated measures, and three-way repeated-measures ANOVA (sex vs gonadal status vs picoamperes, pA). No differences were found in the three-way ANOVA, and data were thus consolidated to look for a main effect of either sex or gonadectomy by two-way repeated-measures ANOVA. For two-way ANOVA, the Bonferroni *post hoc* is considered sufficiently robust to use with non-normally distributed data (Underwood, 1996). The difference of the means (Diff) was defined for SEX (female–male), GDX (sham–castrate), and interaction (GDX/female–SEX/female) – (GDX/male–SEX/male). Significance was set at *p* < 0.05.

## Results

### Verification of gonadectomy

Short-term gonadectomy had no effect on body mass within either sex [females: *n* = 12 mice; sham diestrus, 21.4 ± 0.6 g; *n* = 14 mice; OVX, 21.8 ± 0.3 g; males: *n* = 13 mice; sham, 26.1 ± 0.7 g; *n* = 13 mice; ORX, 25.4 ± 0.6 g; two-way ANOVA gonadal status: *F*_(1,48)_ = 0.07108; *p* = 0.7909; Diff, 0.1500 g (CI, −0.9813, 1.281)]. As expected, males were heavier than females [two-way ANOVA; sex: *F*_(1,48)_ = 53.80; *p* < 0.0001; Diff, −4.127 g (CI, −5.258, −2.996)]. In females, OVX reduced uterine mass [numbers of mice as indicated above; sham diestrus, 63.4 ± 5.0 mg; OVX, 29.6 ± 1.3 mg; unpaired two-tailed Student’s *t* test: *t* = 7.001, df = 24, *p* < 0.0001; Diff, −33.85 ± 4.834 mg (CI, −43.82, −23.87)]. In males, ORX reduced seminal vesicle mass [sham, 217.2 ± 11.6 mg; ORX, 56.6 ± 4.4 mg; unpaired two-tailed Student’s *t* test: *t* = 12.97, df = 24, *p* < 0.0001; Diff, −160.7 ± 12.39 mg (CI, −186.3, −135.1)].

### Passive properties of GnRH neurons do not vary with sex or gonadal status under conditions used to characterize potassium currents

**Figure 1.**
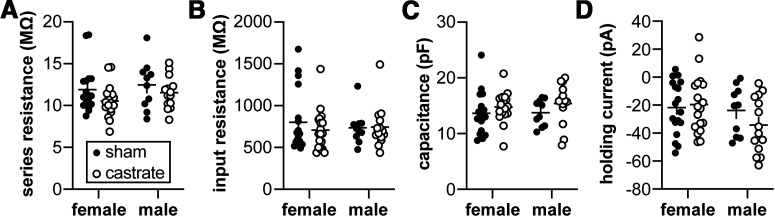
Passive properties of GnRH neurons combined from the potassium current experiments. ***A–D***, No differences attributable to sex or gonadal status were detected in series resistance (***A***), input resistance (***B***), capacitance (***C***), or holding current (***D***) using two-way ANOVA (Table 1). Female sham, *n* = 18 cells, 11 mice; female castrate, *n* = 18 cells, 12 mice; male sham, *n* = 10 cells, 9 mice; male castrate, *n* = 16 cells, 11 mice.

Critical to the evaluation of current properties among treatments is the comparison of similar quality recordings. No difference was observed among groups for uncompensated series resistance (Fig. 1*A*). Nor were any differences in the passive cellular properties of input resistance (Fig. 1*B*), capacitance (Fig. 1*C*), or holding current (Fig. 1*D*) attributable to either sex or gonadal status. These latter measures offer a combined view of recording quality as well as insight into potential biological changes, such as cell size or membrane conductance (two-way ANOVA; Table 1).

**Table 1:** Statistical parameters from two-way ANOVA for passive properties from potassium current recordings (Fig. 1)

Property	Sex	Gonadal status	Interaction
Series resistance (MΩ)	Diff, −0.7697 [CI, −2.024, 0.4842]	Diff, 1.138 [CI, −0.1155, 2.392]	Diff, 0.4283 [CI, −2.936, 2.079]
	*F*_(1,56)_ = 1.512; *p* = 0.2240	*F*_(1,56)_ = 3.308; *p* = 0.0743	*F*_(1,56)_ = 0.1171; *p* = 0.7335
Input resistance (MΩ)	Diff, 14.39 [CI, −136.0, 164.8]	Diff, 42.39 [CI, −108.0, 192.8]	Diff, 101.2 [CI, −199.6, 402.1]
	*F*_(1,56)_=0.03673; *p* = 0.8487	*F*_(1,56)_=0.3186; *p* = 0.5747	*F*_(1,56)_=0.4542; *p* = 0.5031
Capacitance (pF)	Diff, −0.3821 [CI, −2.109, 1.345]	Diff, −1.374 [CI, −3.10, 0.353]	Diff, −0.5171 [CI, −3.972, 2.938]
	*F*_(1,56)_ = 0.1963; *p* = 0.6594	*F*_(1,56)_ = 2.540; *p* = 0.1166	*F*_(1,56)_ = 0.08991; *p* = 0.7654
Holding current (pA)	Diff, 8.334 [CI, −2.035, 18.70]	Diff, 4.110 [CI, −6.259, 14.48]	Diff, 12.66 [CI, −8.074, 33.40]
	*F*_(1,56)_ = 2.592; *p* = 0.1130	*F*_(1,56)_ = 0.6304; *p* = 0.4306	*F*_(1,56)_ = 1.496; *p* = 0.2263

### Neither sex nor gonadal status affect voltage-gated potassium currents of GnRH neurons

**Figure 2.**
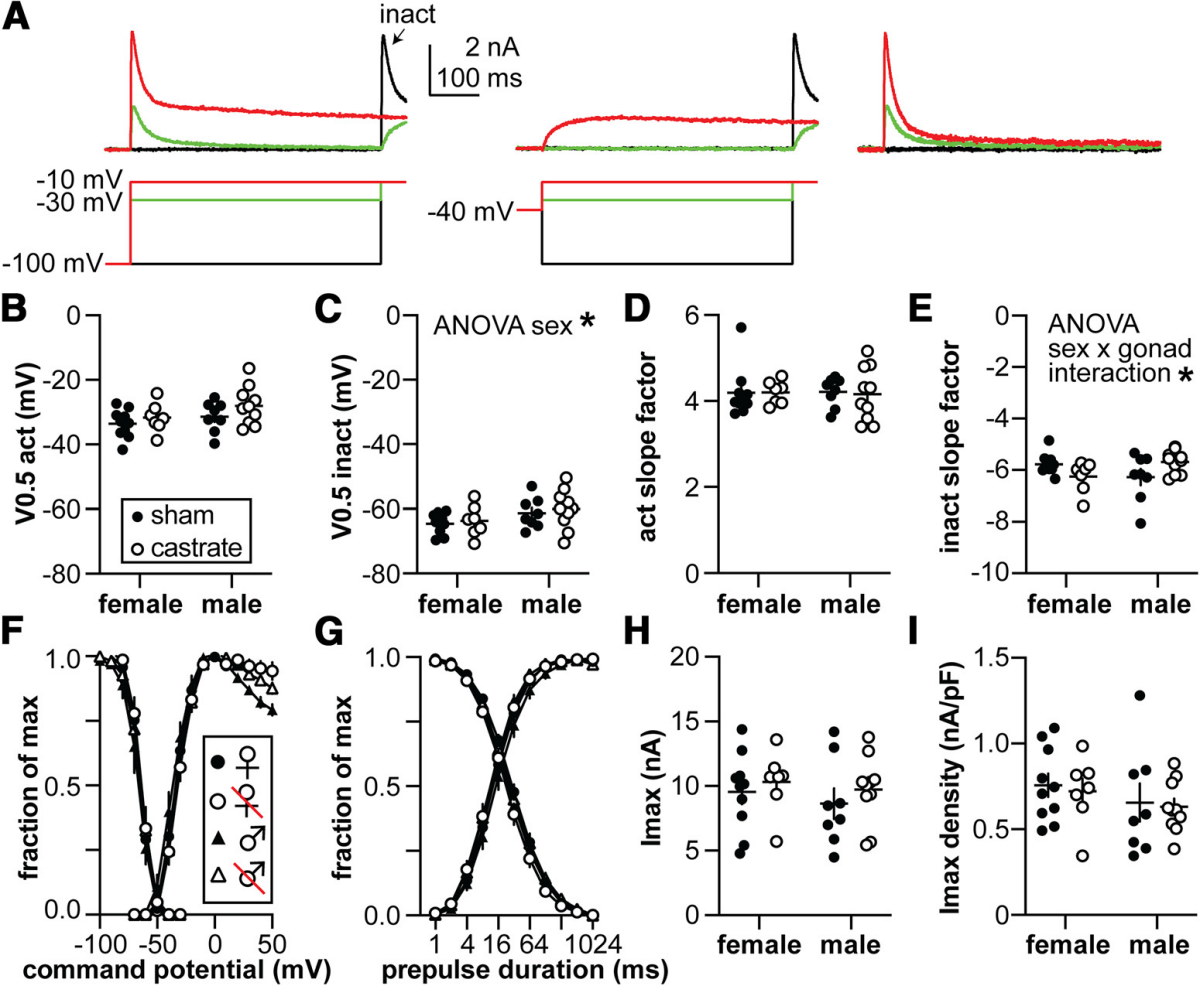
Characterization of the *I*_A_ potassium current. ***A***, Representative traces illustrating the isolation of the rapidly inactivating *I*_A_ (top) and the voltage protocols used (bottom). The 500 ms −100 mV prepulse was truncated for illustration. The arrow labeled “inact” indicates the −10 mV test pulse at the end of the activation voltage family used to calculate the voltage dependence of inactivation. Only three steps from the voltage family from −100 to +50 mV are shown for clarity. The right panel shows *I*_A_ isolated by subtracting the −40 mV prepulse traces in the middle panel from the −100 mV prepulse traces in the left panel. ***B***, ***C***, Membrane potential at which half of the current is activated (*V*_0.5act_; ***B***) or inactivated (*V*_0.5inact_; ***C***). ***D***, ***E***, Activation (act; ***D***) and inactivation (inact; ***E***) slope factors. ***F***, Voltage dependence of activation and inactivation, normalized by maximum conductance and maximum current, respectively. ***G***, Time course of recovery and inactivation, normalized by maximum current. ***H***, ***I***, Maximum current (*I*_max_; ***H***) and current density (***I***). Statistical parameters are in Table 2. Female sham, *n* = 10 cells, 8 mice; female castrate, *n* = 7 cells, 6 mice; male sham, *n* = 8 cells, 6 mice; male castrate, *n* =10 cells, 8 mice.

**Figure 3.**
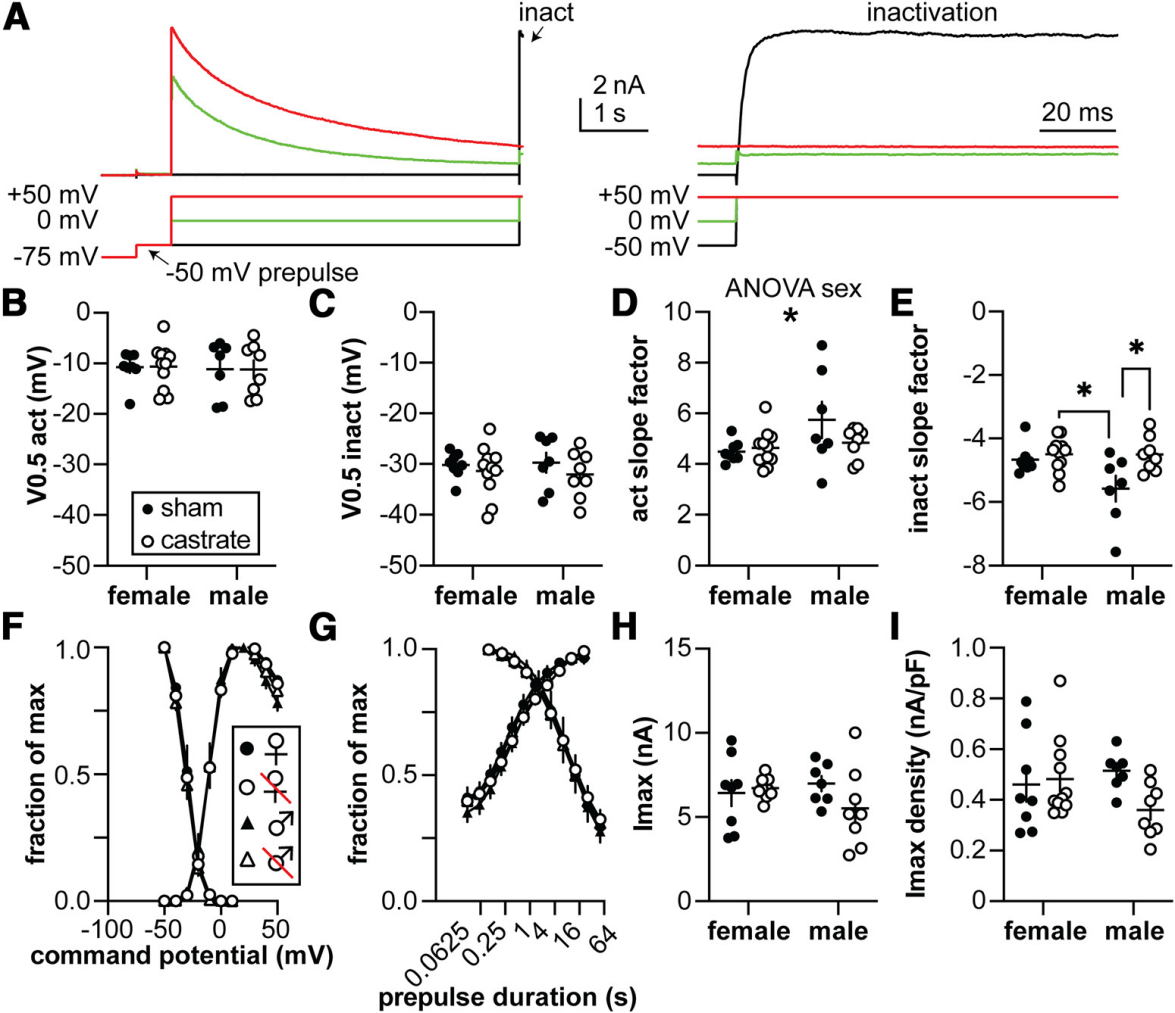
Characterization of the *I*_k_ potassium current. ***A***, Representative traces illustrating the activation and inactivation of *I*_K_, (top) and the voltage protocols used (bottom). The 10 s −75 mV prepulse was truncated in the left panel for illustration. The arrow labeled “inact” on the left panel indicates the region expanded on the right. Only three steps of the voltage family from −100 to +50 mV are shown for clarity. ***B***, ***C***, Membrane potential at which half of the current is activated (*V*_0.5act_; ***B***) or inactivated (*V*_0.5inact_; ***C***). ***D***, ***E***, Activation (act; ***D***) and inactivation (inact; ***E***) slope factors. ***F***, Voltage dependence of activation and inactivation, normalized by maximum conductance and maximum current, respectively. ***G***, Time course of recovery and inactivation, normalized by maximum current. ***H***, ***I***, Maximum current (*I*_max_; ***H***) and current density (Imax density, I); ***I***). Statistical parameters are shown in Table 3. Female sham, *n* = 8 cells, 5 mice; female castrate, *n* = 11 cells, 8 mice; male sham, *n* = 7 cells, 6 mice; male castrate, *n* = 8 cells, 7 mice.

The output of GnRH neurons in terms of spontaneous firing pattern and hormone release is increased by the removal of homeostatic gonadal steroid feedback accomplished via gonadectomy. We hypothesized that removing gonadal steroid feedback would reduce voltage-gated potassium currents. Two components of this current were examined, a fast *I*_A_-like current and a slowly inactivating *I*_K_-like current. No measured property of either current differed with sex or gonadal status (Figs. 2, 3, Tables 2, 3). Only the parameters calculated from the Boltzmann fit, specifically the inactivation *V*_0.5_ and slope factor for *I*_A_, and both the inactivation and activation slope factors for *I*_K_ exhibited *p* values that were just under the value accepted as significant.

**Table 2:** Two-way ANOVA statistical parameters for *I*_A_

Property	Sex	Gonadal status	Interaction
*V*_0.5_ activation (mV)	Diff, −2.916 [CI, −6.395, 0.5635]	Diff, −2.621 [CI, −6.101, 0.8579]	Diff, −1.509 [CI, −8.468, 5.450]
	*F*_(1,31)_ = 2.921; *p* = 0.0974	*F*_(1,31)_ = 2.361; *p* = 0.1345	*F*_(1,31)_ = 0.1956; *p* = 0.6614
*V*_0.5_ inactivation (mV)	**Diff, −3.444 [CI, −6.765, −0.1229]**	Diff, −1.069 [CI, −4.390, 2.252]	Diff, −0.2950 [CI, −6.937, 6.347]
	***F*_(1,31)_ = 4.473; *p* = 0.0426**	*F*_(1,31)_ = 0.4313; *p* = 0.5162	*F*_(1,31)_ = 0.008208; *p* = 0.9284
Activation slope factor	Diff, 11.06 [CI, −339.1, 361.2]	Diff, 23.13 [CI, −327.1, 373.3]	Diff, 52.69 [CI, −647.7, 753.1]
	*F*_(1,31)_ = 0.004146; *p* = 0.9491	*F*_(1,31)_ = 0.01815; *p* = 0.8937	*F*_(1,31)_ = 0.02354; *p* = 0.8791
Inactivation slope factor	Diff, −29.33 [CI, −441.4, 385.8]	Diff, −54.24 [CI, −469.3, 360.8]	**Diff, −1087 [CI, −1897, −236.8]**
	*F*_(1,31)_ = 0.02076; *p* = 0.8864	*F*_(1,31)_ = 0.07103; *p* = 0.7916	***F*_(1,31)_ = 6.872; *p* = 0.0134**
Imax (pA)	Diff, 752.8 [CI, −1252, 2758]	Diff, −935.6 [CI, −2941, 1070]	Diff, −331.7 [CI, −4342, 3697]
	*F*_(1,31)_ = 0.5863; *p* = 0.4496	*F*_(1,31)_ = 0.9055; *p* = 0.3487	*F*_(1,31)_ = 0.02845; *p* = 0.8671
Imax density (pA/pF)	Diff, 95.94 [CI, −59.94, 251.8]	Diff, 30.69 [CI, −125.2, 186.6]	Diff, −12.04 [CI, −323.8, 299.7]
	*F*_(1,31)_ = 1.576; *p* = 0.2188	*F*_(1,31)_ = 0.1613; *p* = 0.6907	*F*_(1,31)_ = 0.006205; *p* = 0.9377

Bold indicates *p* < 0.05.

**Table 3:** Two-way ANOVA statistical parameters for *I*_K_

Property	Sex	Gonadal status	Interaction
*V*_0.5_ activation (mV)	Diff, 0.4790 [CI, −2.780, 3.738]	Diff, −0.03127 [CI, −3.290, 3.227]	Diff, 0.2534 [CI, −6.264, 6.771]
	*F*_(1,30)_ = 0.09013; *p* = 0.7661	*F*_(1,30)_ = 0.0003841; *p* = 0.9845	*F*_(1,30)_ = 0.006304; *p* = 0.9372
*V*_0.5_ inactivation (mV)	Diff, 0.1244 [CI, −3.086, 3.335]	Diff, 1.572 [CI, −1.455, 4.966]	Diff, 1.107 [CI, −5.315, 7.528]
	*F*_(1,30)_ = 0.006266; *p* = 0.9374	*F*_(1,30)_ = 1.247; *p* = 0.2731	*F*_(1,30)_ = 0.1239; *p* = 0.7273
Activation slope factor	**Diff, −729.3 [CI, −1448. 10.51]**	Diff, 380.7 [CI, −338.0, 1099]	Diff, 1043 [CI, −394.3, 2481]
	***F*_(1,30)_ = 4.294; *p* = 0.0469**	*F*_(1,30)_ = 1.170; *p* = 0.2880	*F*_(1,30)_ = 2.197; *p* = 0.1487
Inactivation slope factor	Diff, 459.6 [CI, −11.40, 930.5]	**Diff, −626.4 [CI, −1097, −155.4]**	Diff, −906.0 [CI, −1849, 35.34]
	*F*_(1,30)_ = 3.971; *p* = 0.0554	***F*_(1,30)_ = 7.378; *p* = 0.0109**	*F*_(1,30)_ = 3.864; *p* = 0.0587
Imax (pA)	Diff, 318.3 [CI, −872.6, 1509]	Diff, 588.6 [CI, −602.3, 1780]	Diff, 1753 [CI, −628.8, 4135]
	*F*_(1,30)_ = 0.2979; *p* = 0.5892	*F*_(1,30)_ = 1.019; *p* = 0.3208	*F*_(1,30)_ = 2.259; *p* = 0.1433
Imax density (pA/pF)	Diff, 34.25 [CI, −69.08, 137.6]	Diff, 66.45 [CI, −36.88, 169.8]	Diff, 174.9 [CI, −31.75, 381.6]
	*F*_(1,30)_ = 0.4582; *p* = 0.5036	*F*_(1,30)_ = 1.725; *p* = 0.1990	*F*_(1,30)_ = 2.988; *p* = 0.0942

Bold indicates *p* < 0.05.

### Passive properties of GnRH neurons do not vary with sex or gonadal status under conditions used to characterize action potentials

**Figure 4.**
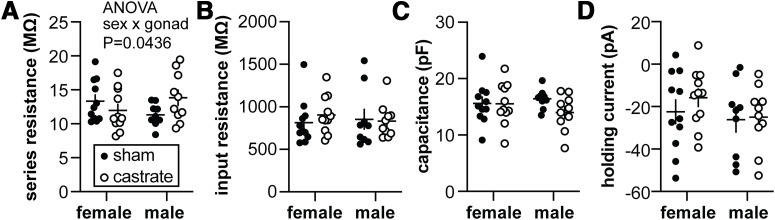
Passive properties of recordings for the action potential study. ***A–D***, Series resistance (***A***), input resistance (***B***), capacitance (***C***), and holding current at −65 mV (***D***). Statistical parameters are in Table 4. Female sham, *n* = 11 cells, 6 mice; female castrate, *n* = 11 cells, 8 mice; male sham, *n* = 9 cells, 6 mice; male castrate, *n* = 10 cells, 6 mice (*no post hoc* comparisons were significant).

To record action potential properties, the blockers of voltage-gated calcium and sodium channels needed to isolate potassium currents were omitted; this may alter the passive properties of the cells, thus these parameters are reported separately for the two types of recordings (Fig. 4, Table 4). There was a weak interaction between sex and gonadal status for uncompensated series resistance (Fig. 4*A*; *p*  = 0.0436). This difference is unlikely to account for a difference in measured values as bridge balance was in effect to compensate series resistance by >95% during action potential recordings. As was observed for recordings of potassium currents, there were no differences in the passive cellular properties of input resistance (Fig. 4*B*), capacitance (Fig. 4*C*), or holding current (Fig. 4*D*) attributable to either sex or gonadal status.

**Table 4:** Two-way ANOVA statistical parameters for passive properties from action potential recordings (**Fig. 5)**

Property	Sex	Gonadal status	Interaction
Series resistance (MΩ)	Diff, 0.06267 [CI, −1.809, 1.935]	Diff, −5.769 [CI, −2.449, 1.295]	**Diff, −3.861 [CI, −7.605, 0.1168]**
	*F*_(1,37)_ = 0.004601; *p* = 0.9463	*F*_(1,37)_ = 0.3899; *p* = 0.5362	***F*_(1,37)_ = 4.366; *p* = 0.0436**
Input resistance (MΩ)	Diff, 16.08 [CI, −149.6, 181.8]	Diff, −35.00 [CI, −200.7, 130.7]	Diff, 109.1 [CI, −222.3, 440.5]
	*F*_(1,37)_ = 0.03866; *p* = 0.8452	*F*_(1,37)_ = 0.1832; *p* = 0.6712	*F*_(1,37)_ = 0.4448; *p* = 0.5089
Capacitance (pF)	Diff, 0.3651 [CI, −1.687, 2.417]	Diff, 1.249 [CI, −0.8033, 3.301]	Diff, 2.347 [CI, −1.757, 6.452]
	*F*_(1,37)_ = 0.1299; *p* = 0.7205	*F*_(1,37)_ = 1.521; *p* = 0.2253	*F*_(1,37)_ = 1.343; *p* = 0.2539
Holding current (pA)	Diff, 6.409 [CI, −4.005, 16.82]	Diff, −3.849 [CI, −14.26, 6.594]	Diff, 5.480 [CI, −15.35, 26.31]
	*F*_(1,37)_ = 1.555; *p* = 0.2202	*F*_(1,37)_ = 0.5610; *p* = 0.4586	*F*_(1,37)_ = 0.2843; *p* = 0.5971

Bold indicates *p* < 0.05.

### Neither excitability nor first action potential properties are altered by sex or gonadal status

**Figure 5.**
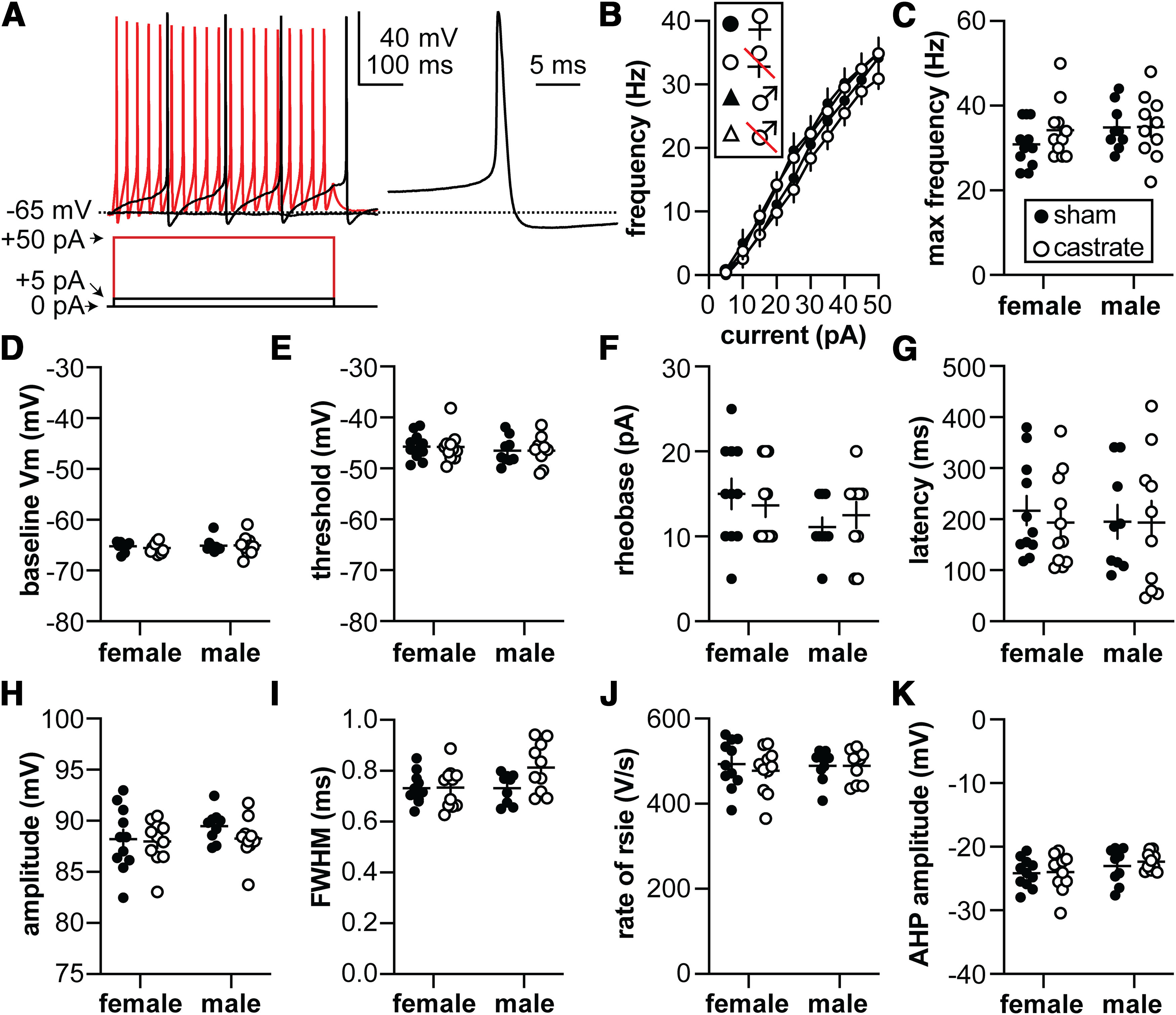
Action potential properties. ***A***, Left, Representative membrane voltage (top) responses to current injections (bottom); only three steps are shown for clarity. Right, Expanded first action potential waveform. ***B***, Frequency of action potentials as a function of current injection. ***C***, Maximum action potential frequency. ***D***, Baseline membrane potential before rheobase current injection. ***E***, Action potential threshold. ***F***, Rheobase (minimum current to produce an action potential). ***G***, Action potential latency. ***H***, Action potential amplitude. ***I***, Action potential FWHM. ***J***, Maximum rate of rise of the action potential. ***K***, Amplitude of the AHP. Note that *y*-axes of ***D***, ***E***, and ***H*** do not start or end at zero. Statistical parameters are shown in Tables 5 and 6. Female sham, *n* = 11 cells, 6 mice; female castrate, *n* = 11 cells, 8 mice; male sham, *n* = 9 cells, 6 mice; male castrate, *n* = 10 cells, 6 mice.

Current-clamp recordings of action potential properties were used to assess possible general differences in the intrinsic properties of GnRH neurons in the experimental groups (Fig. 5). No differences were observed in the firing response to current injection attributable to sex or gonadal status (three-way repeated-measures ANOVA; Table 5). Postanalysis consolidation of these data to examine effects of either sex or gonadal status by two-way repeated-measures ANOVA revealed a mild interaction between sex and current injection, but no *post hoc* significance (Bonferroni test); there was no effect of gonadectomy. There were also no differences observed in any action potential property examined (two-way ANOVA; Table 6). Together, these observations suggest that homeostatic feedback does not induce major changes in action potentials of GnRH neurons.

**Table 5:** Three-way repeated-measures parameters for action potential firing as a function of current injection

	Three way	Two way (sex consolidated)	Two way (GDX consolidated)
Current step (pA)	***F*_(2.554,91.96)_ = 641.8; *p* < 0.0001**	***F*_(10,380)_ = 653; *p* < 0.0001**	***F*_(10,380)_ = 636; *p* < 0.0001**
Sex	*F*_(1,36)_ = 2.447; *p* = 0.1265	*F*_(1,38)_ = 2.6; *p* = 0.1119	
GDX	*F*_(1,36)_ = 0.4993; *p* = 0.4844		*F*_(1,38)_ = 0.64; *p* = 0.4271
pA × sex	*F*_(10,360)_ = 1.758; *p* = 0.0668	***F*_(10,380)_ = 1.9; *p* = 0.0477**	
pA × GDX	*F*_(10,360)_ = 0.7544; *p* = 0.6728		*F*_(10,380)_ = 0.88; *p* = 0.5513
sex × GDX	*F*_(1,36)_ = 0.2066; *p* = 0.6522		
pA × sex × GDX	*F*_(10,360)_ = 0.5547; *p* = 0.8503		

Bold indicates *p* < 0.05.

**Table 6:** Two-way ANOVA parameters for action potential properties

Property	Sex	Gonadal status	Interaction
Max frequency (Hz)	Diff, −2.399 [CI, −6.417, 1.619]	Diff, −1.692 [CI, −5.710, 2.325]	Diff, 3.162 [CI, −4.875, 11.20]
	*F*_(1,37)_ = 1.463; *p* = 0.2341	*F*_(1,37)_ = 0.7278; *p* = 0.3991	*F*_(1,37)_ = 0.6354; *p* = 0.4305
Baseline (mV)	Diff, −0.3300 [CI, 01.182, 0.521]	Diff, 0.1612 [CI, −0.6906, 1.013]	Diff, −0.3928 [CI, −2.096, 1.311]
	*F*_(1,37)_ = 0.6163; *p* = 0.4374	*F*_(1,37)_ = 0.1470; *p* = 0.7036	*F*_(1,37)_ = 0.2182; *p* = 0.6431
Threshold (mV)	Diff, 0.7306 [CI, −1.011, 2.472]	Diff, 0.03322 [CI, −1.709, 1.775]	Diff, −0.8372 [CI, −3.567, 3.400]
	*F*_(1,37)_ = 0.7224; *p* = 0.4008	*F*_(1,37)_ = 0.001493; *p* = 0.9694	*F*_(1,37)_ = 0.002371; *p* = 0.9614
Rheobase (pA)	Diff, 2.513 [CI, −0.5418, 5.567]	Diff, −0.01263 [CI, −3.067, 3.042]	Diff, −2.753 [CI, −8.861, 3.356]
	*F*_(1,37)_ = 2.778; *p* = 0.1040	*F*_(1,37)_ = 7.015e-005; *p* = 0.9934	*F*_(1,37)_ = 0.8335; *p* = 0.3672
AP latency (ms)	Diff, 11.08 [CI, −56.00, 78.15]	Diff, 12.35 [CI, −54.72, 79.43]	Diff, −21.55 [CI, −155.7, 112.6]
	*F*_(1,37)_ = 0.1120; *p* = 0.7398	*F*_(1,37)_ = 0.1392; *p* = 0.7112	*F*_(1,37)_ = 0.1060; *p* = 0.7466
AP amplitude (mV)	Diff, −0.7925 [CI, −2.275, 0.6903]	Diff, 0.7203 [CI, −0.7625, 2.203]	Diff, 1.002 [CI, −1.964, 3.967]
	*F*_(1,37)_ = 1.173; *p* = 0.2858	*F*_(1,37)_ = 0.9688; *p* = 0.3314	*F*_(1,37)_ = 0.4683; *p* = 0.4980
AP FWHM (ms)	Diff, −0.03974 [CI, −0.08772, 0.008232]	Diff, −0.04148 [CI, −0.08945, 0.006499]	Diff, −0.08005 [CI, −0.1760, 0.01589]
	*F*_(1,37)_ = 2.817; *p* = 0.1017	*F*_(1,37)_ = 3.069; *p* = 0.0881	*F*_(1,37)_ = 2.858; *p* = 0.0993
AP rate of rise (mV/ms)	Diff, −4.028 [CI, −34.20, 26.14]	Diff, 7.957 [CI, −22.21, 38.13]	Diff, −16.06 [CI, −76.40, 44.27]
	*F*_(1,37)_ = 0.07317; *p* = 0.7883	*F*_(1,37)_ = 0.2856; *p* = 0.5962	*F*_(1,37)_ = 0.2910; *p* = 0.5928
AHP amp (mV)	Diff, −1.383 [CI, −2.909, 0.1425]	Diff, −0.4236 [CI, −1.949, 1.102]	Diff, 0.5332 [CI, −3.584, 2.518]
	*F*_(1,37)_ = 3.374; *p* = 0.0743	*F*_(1,37)_ = 0.3165; *p* = 0.5771	*F*_(1,37)_ = 0.1254; *p* = 0.7253

## Discussion

A century ago, early studies of pituitary ablation and replacement revealed a link between the emerging science of neuroendocrinology and reproductive processes (Evans, 1921; Smith, 1927). Around the same time, the reverse link was being made by observing the effects of orchidectomy on the pituitary and the amelioration of these effects by replacement with fat-soluble extracts of the testes (McCullagh, 1932). It is now well established that reproduction revolves around the stimulatory and feedback interactions of the hypothalamo–pituitary–gonadal axis, with GnRH neurons serving as the final common pathway for central signaling to the pituitary. These interactions are homeostatic in males and homeostatic throughout most of the reproductive cycle in females; removal of the gonads opens these homeostatic feedback loops and results in increased GnRH neuron activity, GnRH release, and LH release in both sexes (Leipheimer et al., 1985; Levine et al., 1985; Karsch et al., 1987; Caraty and Locatelli, 1988; Condon et al., 1988; Jackson and Kuehl, 2000; Han et al., 2020). Despite the similarity in steady-state endocrine response, it is possible that latent sex differences in underlying neurobiological mechanisms exist (Jain et al., 2019). We tested the hypotheses that the removal of negative homeostatic feedback would reduce potassium currents and increase the excitability of GnRH neurons. Based on the parameters we quantified, minimal differences were observed that could be attributed to sex or feedback condition. We thus reject these hypotheses.

In our analysis of potassium currents, a small difference was detected in the calculated membrane potential at which half of the channels conducting *I*_A_ are inactivated (*V*_0.5inact_). When this value is depolarized (value closer to zero), less current would be available and vice versa. In GnRH neurons, this value is similar to their estimated baseline membrane potential (Berg et al., 2018). The *V*_0.5inact_ was more depolarized in males, meaning less *I*_A_ is available at baseline membrane potential. The physiological relevance of the −3.4 mV shift can be interpreted from the action potential latency in the current-clamp recordings, a parameter we previously linked to changes in *I*_A_ (DeFazio and Moenter, 2002). The lack of difference in AP latency (or any other AP parameter tested) suggests minimal physiological relevance of this shift. Similarly, the differences in slope factors for *I*_A_ and *I*_K_ do not appear to affect action potential firing. Thus, while these values are statistically different, the biological impact of these minimal differences appears insufficient to alter action potential firing under the conditions tested.

The present data showing minimal changes in the intrinsic properties of GnRH neurons in females between diestrous and OVX mice support prior work assessing the excitability of GnRH neurons in OVX versus OVX+E mice exhibiting diurnal changes between negative and positive feedback (Christian et al., 2005). In this model, GnRH neuron activity and LH release are suppressed in the morning in OVX+E mice relative to OVX mice, demonstrating homeostatic negative feedback, but increased in the afternoon in cells from OVX+E mice, demonstrating estradiol-positive feedback. Interestingly, there were no differences in GnRH neuron excitability among OVX mice studied in the morning or afternoon and OVX+E mice in the morning (negative feedback), whereas GnRH neurons from OVX+E mice recorded in the afternoon during positive feedback were more excitable (Adams et al., 2018b). The present work extends these data to include both a comparison between the sexes and a comparison using the open-loop OVX/ORX condition versus sham-operated controls to ameliorate caveats associated with steroid hormone replacement. The lack of effect of removing homeostatic feedback suggests that a majority of the changes that lead to increased GnRH output in gonadectomized animals are processed presynaptically to these cells. In and of itself, this concept is not new as GnRH neurons do not appear to express detectable levels of steroid hormone receptors other than the β-isoform of the estradiol receptor (Herbison et al., 1996; Hrabovszky et al., 2000, 2007). There has thus long been a relative consensus in the field that steroid feedback is integrated via upstream, steroid-responsive cells (Wintermantel et al., 2006). The lack of observed changes in GnRH neuron properties examined suggests that homeostatic negative feedback signals are conveyed from these afferents in a manner that alters the output of GnRH neurons without substantial biophysical changes at the level of the cell soma. Of note, there is increased frequency of GABA_A_ receptor-mediated postsynaptic currents, which can excite GnRH neurons (DeFazio et al., 2002; Herbison and Moenter, 2011), observed in GnRH neurons from ORX versus intact males (Chen and Moenter, 2009) and in cells from OVX versus OVX+E females during negative feedback (Christian and Moenter, 2007). Postsynaptic currents have relatively short-lived effects on membrane potential that may not lead to changes in overall excitability. Interestingly, changing homeostatic negative feedback does engage mechanisms affecting excitability in arcuate kisspeptin neurons, also known as KNDy neurons for their coexpression of the following three neuropeptides: kisspeptin, neurokinin B, and dynorphin (Oakley et al., 2009; Moore et al., 2018). KNDy neurons have been postulated to be key afferent inputs to GnRH neurons to drive episodic release from these cells (Qiu et al., 2016; Clarkson et al., 2017; Vanacker et al., 2017). In KNDy neurons from OVX+E versus OVX females examined during negative feedback in the morning, estradiol reduced fast transient *I*_A_ (DeFazio et al., 2019), and *I*_A_ modifies action potential patterns in these cells (Mendonça et al., 2018).

The lack of changes in the homeostatic versus open-loop models studied in the present work stand in marked contrast to changes that occur in GnRH neurons from females during estradiol-positive feedback (Adams et al., 2018a, b, 2019). In addition to the increase in GnRH neuron excitability observed during the positive feedback mentioned above, GnRH neurons exhibit reduced transient potassium currents and increased high voltage-activated calcium conductances in OVX+E mice during positive feedback (DeFazio and Moenter, 2002; Sun et al., 2010). While both the removal of negative feedback by gonadectomy and the induction of positive feedback both increase GnRH release, the nature of this increase is quite different. Removal of negative feedback maintains the episodic GnRH/LH release pattern that is characteristic in males and most of the reproductive cycle in females (Leipheimer et al., 1985; Levine et al., 1985; Karsch et al., 1987; Caraty and Locatelli, 1988; Condon et al., 1988; Jackson and Kuehl, 2000; Han et al., 2020). Pulse frequency and amplitude are typically increased. Even in long-term castrated rams, when the pulsatile nature of LH release is no longer evident, high-frequency GnRH pulses are clearly observed (Caraty and Locatelli, 1988). The induction of positive feedback, however, shifts the pattern from episodic to a continual elevation above baseline that lasts for several hours (Sarkar et al., 1976; Levine and Ramirez, 1982; Clarke et al., 1987; Moenter et al., 1990, 1991; 1992a,b; Xia et al., 1992). Given the fundamental differences in how the output of GnRH neurons is altered in these two circumstances, it is reasonable to postulate that more extensive changes are required for successful positive feedback, and that this is in part accomplished by extending the mechanisms engaged to the alteration of GnRH neuron intrinsic properties to allow continuous secretion to be maintained. Conceptually, pulse frequency can be rapidly modulated by homeostatic perturbances that influence reproduction. For example, inflammatory stress (Battaglia et al., 1998), hypoglycemia (Chen et al., 1996), and naloxone antagonism of opiates (Caraty et al., 1987) all rapidly induce changes in the pulse pattern of GnRH or multiunit activity associated with LH in the hypothalamus. In contrast, estradiol induction of positive feedback is a process with a substantial (typically, >12h) delay to the increased release, and this increase appears to be all or none as it is not dependent on the continued presence of estradiol (Evans et al., 1997).

The apparently different postsynaptic effects of removing negative feedback and inducing positive feedback raise some interesting questions for future contemplation. This is particularly true given that the prevailing view in the field is that the neuromodulator kisspeptin plays an important role in activating both episodic and surge modes of GnRH release (Porteous and Herbison, 2019; Wang et al., 2019). Kisspeptin application in brain slices reduces *I*_A_ in a manner similar to the induction of estradiol-positive feedback (Pielecka-Fortuna et al., 2011) and also increases the excitability of GnRH neurons (Adams et al., 2018b), indicating it can alter the intrinsic properties of these cells. The two hypothalamic populations of kisspeptin neurons, the aforementioned KNDy neurons in the arcuate and those in the anteroventral periventricular area are thought to mediate pulsatile and surge modes of GnRH release, respectively. These populations possess different cotransmitters and mediators (Cravo et al., 2011; Skrapits et al., 2015); if co-released with kisspeptin, these could produce counteracting effects in the postsynaptic GnRH neuron. Alternatively, other afferent populations may have critical roles. In this regard, the increase in GnRH neuron firing induced by kisspeptin application is longer in duration than the typical pulse (Moenter et al., 1992b; Evans et al., 1996; Han et al., 2005; Pielecka-Fortuna et al., 2008). The neuromodulator known as gonadotropin inhibitory hormone in birds and RFRP3 in mammals can counteract the activating effects of applied kisspeptin on GnRH neuron firing rate in brain slices by activating a potassium current (Wu et al., 2009). While speculative, an interplay of kisspeptin and RFRP3 may have opposing effects on intrinsic GnRH properties.

While we have confidence in the lack of effect of sex or removing homeostatic feedback on the properties examined in the present work, it is important to acknowledge alternative possibilities and mechanisms. It is possible that removing negative feedback induces equal and opposite changes in intrinsic properties resulting in masking of these changes in the current-clamp recordings. This seems unlikely given the different kinetic properties of the voltage-gated channels likely to mediate changes in action potential firing and properties. It is also possible that changes are induced in the biophysical properties of GnRH neurons that are distal to the soma and not possible to monitor in our brain slice preparation. The median eminence has long been postulated to be important for episodic release. GnRH release from isolated rat median eminences is pulsatile (Rasmussen, 1993), and release in this region can be affected by different mechanisms. For example, arcuate kisspeptin neurons can interact with GnRH neurons via kisspeptin and neurokinin B at the level of the terminals in the median eminence (Gaskins et al., 2013; Glanowska and Moenter, 2015; Yip et al., 2015). Recent work using chemogenetic or optogenetic approaches showed that targeting this region with G_i_-coupled receptors or archaerhodopsin reduced, but did not eliminate, pulsatile LH release *in vivo* (Wang et al., 2020).

Examining potassium currents and action potential properties at a different point postgonadectomy may have revealed sex differences. While both males and females increase GnRH and LH release following gonadectomy, the increase is somewhat delayed in females. At the time point we investigated of 5–7 d postgonadectomy, clear increases in LH have occurred in both sexes (Yamamoto et al., 1970). It is also possible that removing homeostatic feedback alters other aspects of GnRH neurons to increase GnRH output, such as increasing GnRH mRNA (Finn et al., 1998; Gore, 1998), altering ionotropic receptors that produce brief changes in membrane potential, or changing excitation secretion coupling to make it more effective. Any of these alternative mechanisms could be sexually differentiated.

In sum, the present work rejected the hypotheses that removing homeostatic gonadal feedback induces changes in potassium currents and excitability of GnRH neurons. In so doing, we provide evidence for different mechanistic strategies to regulate the output of GnRH neurons during homeostatic versus positive feedback.

## Synthesis

Reviewing Editor: Rae Silver, Columbia University Decisions are customarily a result of the Reviewing Editor and the peer reviewers coming together and discussing their recommendations until a consensus is reached. When revisions are invited, a fact-based synthesis statement explaining their decision and outlining what is needed to prepare a revision will be listed below. The following reviewer(s) agreed to reveal their identity: NONE.

Overview: The work described in this manuscript advances the field by demonstrating that gonadal steroid feedback may not alter the electrical activity of GnRH neurons by altering their intrinsic electrical properties.

More specifically, the authors report on gonadotropin-releasing hormone (GnRH) neuron excitability and voltage gated potassium currents in intact and gonadectomized male and female mice. Three questions were explored. First, because estradiol administered in vivo upregulates fast and slow inactivating potassium currents in GnRH neurons from ovariectomized (OVX) females, the authors hypothesized that removing gonadal steroid negative feedback through gonadectomy would alter these voltage-gated potassium currents. Second, because in OVX female’s estradiol positive feedback, which causes increases in LH secretion, result in increased GnRH neurons excitability (as assessed by the number of action potential fired in response to a depolarizing current), the authors hypothesized that gonadectomy, which also results in increased LH secretion by removing negative feedback, would increase GnRH neuron excitability. Third, as the positive feedback effects of estradiol are female specific in rodents, the authors also tested the hypothesis that negative feedback mechanisms might be sex dependent.

To test these hypotheses, the authors used patch clamp electrophysiology to measure GnRH neuron basic electrophysiological properties, fast inactivating (IA) and slow inactivating (IK) potassium current magnitude and activation/inactivation properties, and GnRH neuron excitability and action potential properties, in 4 groups of mice: intact and gonadectomized male mice and in diestrous and OVX female mice.

The results revealed no overall differences in potassium current characteristics, excitability or action potential properties. The authors, therefore, rejected their hypotheses and concluded that negative feedback effects on the activity of GnRH neurons must be mediated indirectly by afferent neurons, seeing that gonadectomy does not affect GnRH neuron intrinsic properties. In addition this work reveals a lack of sex differences in voltage-gated potassium currents and excitability in GnRH neurons.

Major comments:

1.Consider the following suggestion: The rational and importance of the study might be made much clearer to the reader if it were placed into the background of more recent literature. Today’s facts are that GnRH pulses are driven by KNDy neurons and that the negative feedback is probably largely happening via KNDy neurons. A recent study showed that transiently blocking GnRH neuron firing does not impair GnRH pulses - bringing forth the role of KNDy as the driving force and the moderate role of the GnRH neuron firing pattern for pulses.

2.The authors should comply with eNeuro guidance on presentation of statistical information and consider the use of estimation statistics. The authors are urged to revise their manuscript to include an accurate description of their data and statistical analyses, and to fully utilize the tables and figures provided (table 4 is not even referred to in the text).

Some of the differences are qualified as “weakly significant P values” and “weak” or “mild interactions” in the results section. This would likely help tremendously in the interpretation of the results and would likely help in the interpretation of the data so that phrases such as “weakly significant P values” and “weak” or “mild interactions” in the results section would not be needed.

The results section is insufficiently explained. The authors do not provide any values or sample sizes (numbers of cells or numbers of mice). For example, it is a pity that the study of voltage gated potassium currents, which must have taken weeks to complete, is described in a single sentence stating that “No measured property of either current differed with sex or gonadal status.” Furthermore, the differences in V0.5 inactivation and slope factor of inactivation are not as straightforward as is summarized lines 242-243, and should be detailed.

Minor comments

- The title could better reflect the conclusions of these studies, at least as they are presented in the manuscript.

-The introduction presents the question in a clear way, although the rationale for comparing males and females can be more compelling.

- The authors might consider commenting on the findings in the context of their functional or physiological significance for GnRH neuron firing.

- Do the authors have any data on the resting membrane potential of these cells in the four groups they studied? Did they record GnRH neuron membrane responses to hyperpolarizing currents (depolarizing sag and rebound depolarization)? If so, could these be reported as well?

- Abstract line 16: “... no changes were observed in most potassium current properties...” This statement might need to be clarified: are there no differences between intact and gonadectomized animals or between males and females, or neither?

- Line 101: “variation within a mouse was not less than that within a group.” Was this formally quantified? If so, please describe. If not, please revise.

- Lines 112-113: Although it is unlikely that this would have any effect on what is reported here, a sampling rate 2.5x the cut-off frequency of the low pass filter is recommended to avoid aliasing.

- Line 129: That recordings with series resistance >20 MOhms were discarded is already stated line 117.

- Line 212: In the statistics section, the three way ANOVA analysis is not described and the procedure (and rationale) for the “consolidation” two-way ANOVAs not explained.

- line 241: Tables 2 and 3?
